# Characterization of Rhodamine-123 as a Tracer Dye for Use In *In vitro* Drug Transport Assays

**DOI:** 10.1371/journal.pone.0033253

**Published:** 2012-03-28

**Authors:** Samantha Forster, Alfred E. Thumser, Steve R. Hood, Nick Plant

**Affiliations:** 1 Faculty of Health and Medical Sciences, Centre for Toxicology, University of Surrey, Guildford, Surrey, United Kingdom; 2 PCD DMPK, GlaxoSmithKline, Ware, Hertfordshire, United Kingdom; Biological Research Centre of the Hungarian Academy of Sciences, Hungary

## Abstract

Fluorescent tracer dyes represent an important class of sub-cellular probes and allow the examination of cellular processes in real-time with minimal impact upon these processes. Such tracer dyes are becoming increasingly used for the examination of membrane transport processes, as they are easy-to-use, cost effective probe substrates for a number of membrane protein transporters. Rhodamine 123, a member of the rhodamine family of flurone dyes, has been used to examine membrane transport by the ABCB1 gene product, MDR1. MDR1 is viewed as the archetypal drug transport protein, and is able to efflux a large number of clinically relevant drugs. In addition, ectopic activity of MDR1 has been associated with the development of multiple drug resistance phenotype, which results in a poor patient response to therapeutic intervention. It is thus important to be able to examine the potential for novel compounds to be MDR1 substrates. Given the increasing use rhodamine 123 as a tracer dye for MDR1, a full characterisation of its spectral properties in a range of *in vitro* assay-relevant media is warranted. Herein, we determine λmax for excitation and emission or rhodamine 123 and its metabolite rhodamine 110 in commonly used solvents and extraction buffers, demonstrating that fluorescence is highly dependent on the chemical environment: Optimal parameters are 1% (v/v) methanol in HBSS, with λex = 505 nm, λem = 525 nm. We characterise the uptake of rhodamine 123 into cells, via both passive and active processes, and demonstrate that this occurs primarily through OATP1A2-mediated facilitated transport at concentrations below 2 µM, and via micelle-mediated passive diffusion above this. Finally, we quantify the intracellular sequestration and metabolism of rhodamine 123, demonstrating that these are both cell line-dependent factors that may influence the interpretation of transport assays.

## Introduction

**Figure 1:**
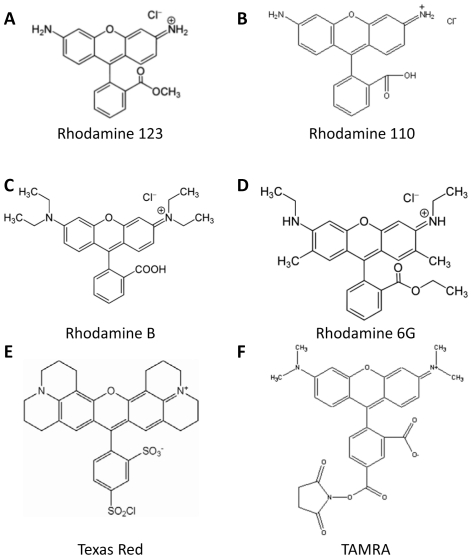
Rhodamine-based flurone dyes.

Within the last decade there has been an increased need for the development of reporter dyes that can be used to examine biological processes within the cellular context. This need is driven by the ability of such dyes to allow the real-time monitoring of cellular processes with regard to both sub-cellular localisation, biological functionality and kinetic parameters [1]; such data can then be used for both *in vitro* and *in silico* mechanistic studies [2], [3], [4], [5], as well as the optimisation of rapid drug screening assays [6]. One such group of tracer dyes are the rhodamine family of flurone dyes, which have multiple applications in fluorescence microscopy, flow cytometry, fluorescence correlation spectroscopy and ELISA. Commonly used flurone dyes include rhodamine-123 (R123), Rhodamine B and Rhodamine 6G, as well as the further modified carboxytetramethylrhodamine (TAMRA), tetramethylrhodamine (TMR) and Texas Red (figure 1). Within this family, R123 has been used extensively as both an inhibitor of mitochondrial function [7], [8] and a tracer for membrane transport [9], [10], [11]. R123 has many advantages as a biological tracer, including commercial availability, low cost, high quantum yield, non-invasive detection and low interference with underlying metabolic processes [8]. However, this utility comes with a number of caveats, such as the observation that R123 fluorescence deviates from linearity above 5 µM in aqueous solution, with the intensity diminishing toward zero at higher concentrations [7]; indeed, R123-seqestration into membranes *in vitro* has been suggested to further reduce the upper limit of linearity to 1 µM [7]. It is therefore important to fully characterize the spectroscopic properties of R123 under a range of commonly used *in vitro* assay conditions to ensure optimal utility of this important tracer dye within *in vitro* transport assays.

The requirement for an accurate quantification of the transport of chemicals across biological membranes has come increasingly to the fore, with the important role of membrane transport proteins being highlighted [12]. This transport impacts upon the absorption, distribution and excretion processes directly, and indirectly determine metabolic fate through the control of chemical access to intracellular metabolic enzyme systems. The protein-meditated transport of drug-like chemicals across biological membranes is predominantly undertaken by the solute carrier (SLC) and ATP-binding cassette (ABC) superfamilies of proteins [13]. SLC family members encompass both uptake and efflux transporters, whereas ABC transporters are exclusively efflux transporters in mammals, with both families utilising energy to transport substrates against their concentration gradient [13]. Together, these drug transport proteins are important determinants of chemical pharmacokinetics [14], [15], having a large impact on intracellular concentrations, and hence biological response. In addition to this important role in normophysiology, atopic expression of drug transport proteins is observed in many tumours [16], [17]; for example, increased expression of MDR1 is associated with a reduced response to therapy in breast cancer [18], presumably due to increased efflux of chemotherapeutic agents from the tumour cells. Given the need to better understand, and quantify, transport processes, tracer dyes are being increasingly used to examine these processes, with R123, Fluo-3 and carboxydichlorofluroscein being increasingly used to study MDR1-, OATP1B3- and MRP2- mediated transport, respectively [4], [19], [20]. Herein, we examine the physiochemical properties of R123, and characterize the optimal conditions for its use as a tracer dye to quantify MDR1-mediated transport. This analysis includes the characterization of optimal emission and excitation wavelengths for R123; the effect of common solvents used for *in vitro* assays; the stability or R123 (both photo- and metabolic), and the membrane transport characteristics. Together, these data demonstrate the optimal conditions for use of R123 as a tracer dye *in vitro* to quantify MDR1-mediated drug efflux. In addition, such work will facilitate the further development of these assays, which are important to fully understand the intracellular pharmacokinetics of administered therapeutics.

## Results and Discussion

### Excitation and Emission Characteristics of R123

**Figure 2:**
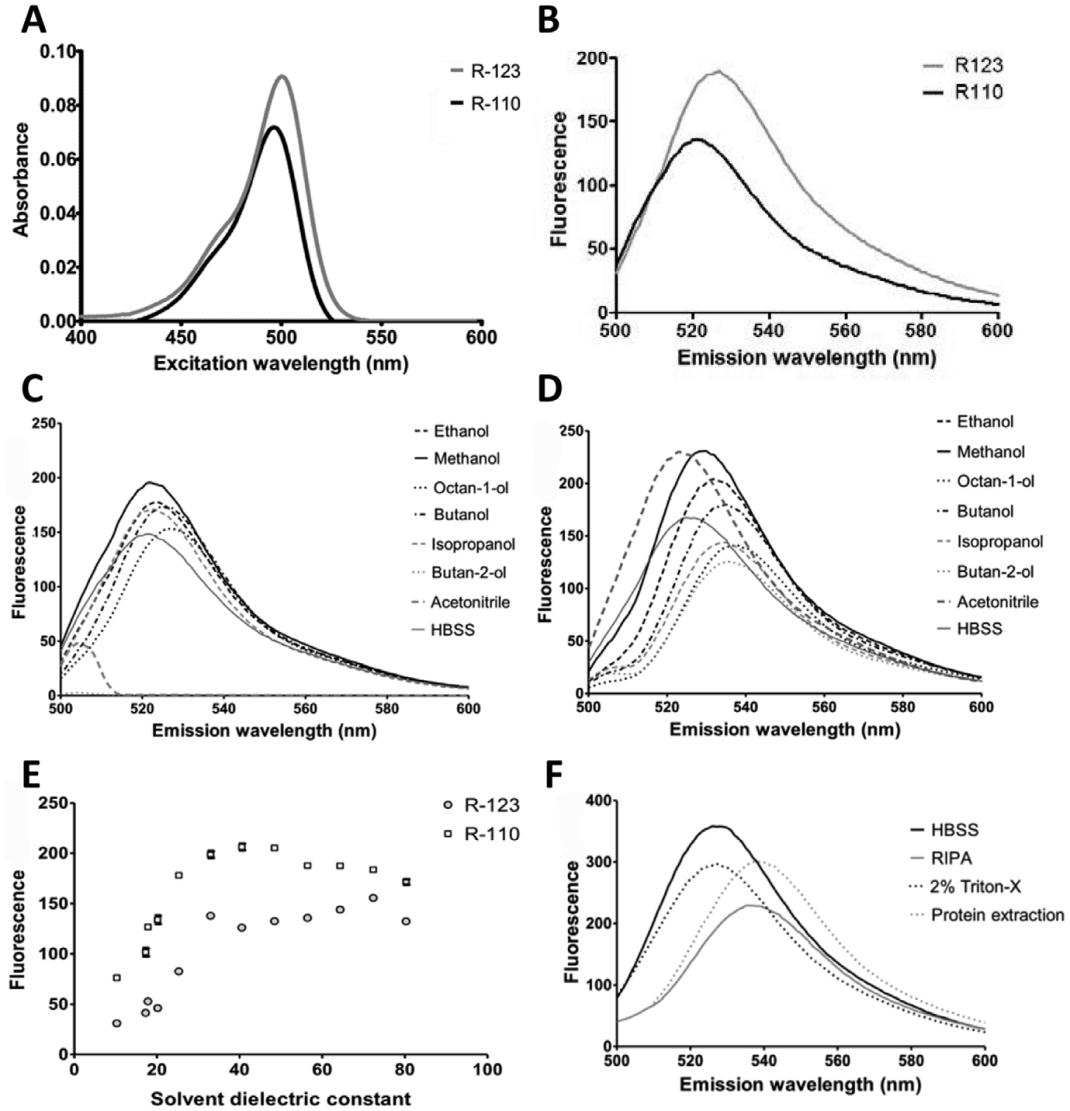
Spectroscopic characteristics of R123 and R110. 1 µM R123 or R110 were prepared in 1% (v/v) MeOH:HBSS. (A) λmax for excitation of each fluorophore was determined using a wavelength absorbance scan (400–600 nm), and (B) λmax for emission determined using a wavelength scan (500–600 nm) with a fixed λex = 505 nm. (C+D) 1 µM R123 or R110 in 1% (v/v) indicated sovent:HBSS were examined with an emission wavelength scan (500–600 nm) with a fixed λex = 505 nm for (C) R123 and (D) R110. (E) Quantum yield for R123 was determined for each solvent combination and plotted against solvent dielectric constant. (F) The impact of commonly used extraction buffers on R123 quantum yield was determined by addition of 1 µM R123 to extraction buffers, followed by an emission wavelength scan (500–600 nm) undertaken with a fixed λex = 505 nm. All data are representative of at least three independent repeats.

In order to accurately assess the levels of both R123 and its primary metabolite R110 we first determined the excitation (λex) and emission (λem) maxima for both compounds. 1 µM R123 and R110 solutions prepared in 1% (v/v) methanol in Hank's buffered saline solution (HBSS), produced maxima absorbance values of 502 nm and 497 nm respectively (Figure 2a): As the R123 certificate of analysis suggests a λex (MeOH) of 507 nm (Sigma-Aldrich, Poole, UK), we selected an intermediate of 505 nm for all subsequent experiments. Using λex = 505 nm, emission spectra were obtained for R123 and R110 under the same solvent composition, determining maxima λem of 525 nm and 520 nm for R123 and R110 emission, respectively (figure 2b). Following these initial experiments, all subsequent experiments were undertaken using 1% (v/v) methanol in HBSS as the medium, and λex = 505 nm, λem = 525 nm unless otherwise stated. It is interesting to note that under these conditions, R123 exhibited both higher absorption and emission intensities than R110 when prepared as equimolar solutions, consistent with the work of Jager and colleagues [21].

It is well established that the local chemical environment may significantly impact on the spectroscopic properties of a flurophore [22], and hence we next investigated the impact of commonly used solvents on fluorescence emmission. 1 µM R123 and R110 solutions were prepared in 1% (v/v) solvent in HBSS, using a variety of analytical grade laboratory solvents. Figure 2c and 2d demonstrate the impact of these solvents on λem for R123 and R110, respectively. In general, all other solvents produced a lower quantum yield than methanol, with this effect more pronounced for R110. In addition, the maxima for λem also differed slightly depending upon the solvent, although this was again more pronounced for R110 than R123. To further examine the relationship between chemical environment and quantum yield, we examined the correlation between solvent dielectric constant and the quantum yield at the λem maxima for each solvent (figure 2e). Quantum yield for both R123 and R110 increases linearly with solvent dielectric constant until ε≈30, whereupon no further increases in quantum yield are observed. As methanol has a dielectric constant of 33, this is consistent with its selection as the solvent of choice for R123. It should be noted that methanol exhibits a dose-dependent toxicity on MDCKII cells, but that this does not reach statistical significance until concentrations in excess of 6% (data not shown). Therefore, at the dose used within subsequent *in vitro* assays (1%), it is possible to produce optimal spectral characteristics without significant cell death.

Given the impact of local chemical environment on the quantum yield of R123, it is also important to consider other commonly used chemicals that may impact upon chemical environment, such as cell lysis solutions. We examined the impact of three commonly used cell lysis buffers, RIPA, PEB and 2% Triton ×100. All three buffers reduced the quantum yield of R123, with RIPA buffer causing a 30% decrease (Figure 2f). In addition, RIPA and PEB caused a significant right shift in λmax, with only triton-×100 retaining the λmax observed with HBSS alone: Based upon these experiments, triton ×100 would seem to be the most sensible lysis buffer if post-lysis fluorescent measurements are required.

### Passive diffusion of R123 across biological membranes

For *in vitro* assays using tracer dyes, the external (loading) concentration used is critical. For efflux assays, the concentration must be sufficient to allow sufficient dye uptake to optimise assay sensitivity. In addition, for both efflux and uptake assays, it is important to understand the relationship between external concentration and the ration of passive and active components of transport. R123 has been reported to cross membranes easily due to its lipophilic nature and accumulate in areas with negative membrane potentials, such as within the matrix of mitochondria [5], [7]. However, a contrary finding *in vitro* is that baseline diffusion from apical to basolateral membranes in Caco-2 cells within transwell culture is negligible [23], which would argue against significant passive diffusion occurring in the cellular context. As increasing evidence suggests that protein-mediated transport processes occur even for those chemicals initially thought to cross membranes solely through passive diffusion [12] it is likely that for R123 there is a mixture of these two processes occurring.

To deconvolute the active and passive components of R123 membrane transport, we first examined the propensity for R123 to passively diffuse by determining the partition coefficient in a simple water:octan-1-ol system. Over the concentration range tested (10–500 nm R123) between 69 – 51% of R123 partitioned into the octan-1-ol phase, with octan-1-ol preference decreasing with increasing R123 concentration (data not shown). At physiological pH, such partition coefficients results in a LogD_7.4_ value of approximately 1.5, which is consistent with previous estimates [24], [25]. In general, passive permeability increases linearly with logD, with Lipinski's rule of five stating that optimal absorption is achieved with a logD value greater that five [26]. Hence, the partitioning estimate derived herein and elsewhere is supportive of only minimal passive diffusion of R123 at the concentrations examined.

**Figure 3:**
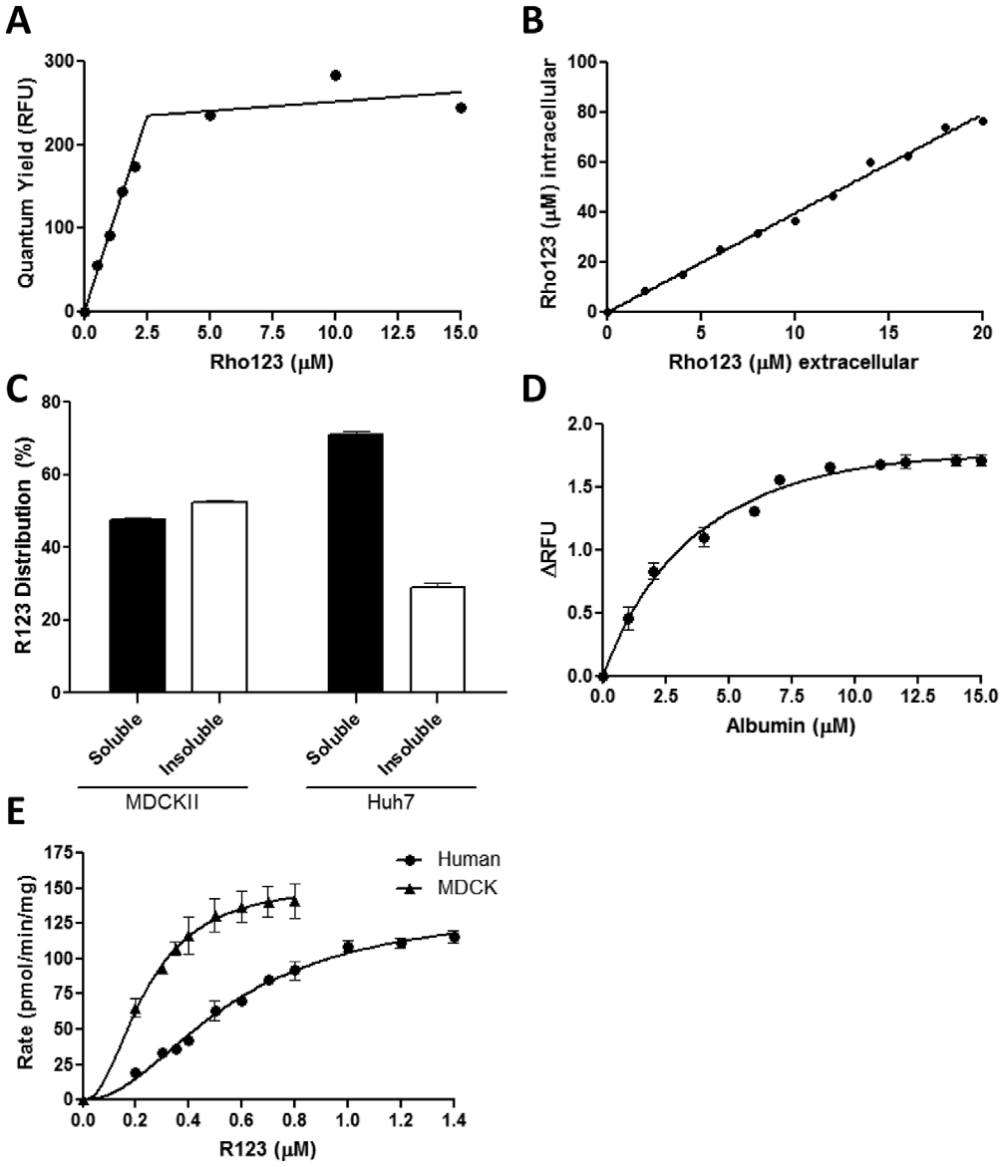
Passive uptake and sequestration of R123. (A) Critical micelle concentration of R123 was determined by measuring quantum yield (λex = 505 nm, λem = 5251nm) of 0–0.5 µM R123 in 1% (v/v)MeOH:HBSS. (B) MDCKII-ABCB1 cells were incubated with 0–20 µM R123 for 10 minutes and intracellular R123 concentration determined against a standard curve following cell lysis with Triton ×100. (C) MDCKII or Huh7 cells were incubated with 10 µM R123 for 10 minutes and R123 concentration in soluble and insoluble cellular fractions determined against R123 standard curves. (D) Albumin binding was determined through the measurement of 0.1 µM R123 fluorescence following the addition of increasing quantities of 0–15 µM albumin. (E) S9 metabolic fractions were extracted from human liver samples and MDCKII cells and used at a final concentration of and respectively. The rate of conversion of R123 to R110 was determined following addition of 0.1 mg/ml or 1 mg/ml S9 fraction from MDCKII cells or human liver, respectively. Rate of conversion was determined for the indicated range of R123, and fitted using an allosteric sigmoidal model of enzyme kinetics. Data points are the average of three separate repeats ± S.D; where no error bars are observed, they are contained within the limits of the data point.

Regardless of the passive permeability of an individual chemical molecule, once chemical concentration exceeds the critical micelle concentration (CMC) then the chemical will aggregate into micelles. From this point on, and almost all additional chemical introduced to the system forms/enters into micelles. Micelles display significantly different transport and metabolic capacities compared to free chemical, and generally exhibit much increased passive permeability across biological membranes [27]. as micelle formation in fluorophores is associated with a shift in λmax and/or altered quantum yield [28], we determined the CMC for R123 through measurement of the fluorescence emission of 0–15 µM R123 concentration. A segmented linear regression of this relationship identifies the CMC concentration as 1.9±0.1 µM (Figure 3a). Once R123 concentration surpasses the CMC, micelles will rapidly form, and these will passively diffuse through the membrane. Hence, for rapid loading of *in vitro* cell systems, a loading concentration in excess of 2 µM would seem optimal. Given that R123-dependent cell toxicity is not significant until concentrations in excess of 20 µM (data not shown), the use of 2 µM as a loading concentration would have no negative impact on cell viability.

It is important to note that the total concentration of R123 within cells is higher than the concentration in the loading medium, by approximately 4-fold (figure 3b), although this is still five-fold lower than the concentration demonstrated to elicit overt toxicity. This disparity is due to sequestration of R123, leading to a heterogeneous sub-cellular localisation [7]. The degree of intracellular sequestration is cell line dependent, with MDCKII cells showing considerably more sequestration than in Huh7 cells, being 50% and 30%, respectively (figure 3c). R123 that is sequestered into membranes or sub-cellular organelles may still be effluxed through active transport mechanism. However, before efflux can occur, the chemical must first diffuse out of the sequestering body. As demonstrated by Yu et al. this can have a significant impact on the overall sub-cellular kinetics and should be considered when calculating accurate kinetic parameters [29]. Determination of the degree of sub-cellular sequestration is, therefore, an important consideration when optimising a novel cell line for quantification of efflux transport.

In addition to the potential for tracer dyes to become sequestered within sub-cellular organelles, it is important to consider components of the medium, or blood *in vivo*, to which tracer dyes may become bound. Many small molecules bind reversibly to albumin and other serum proteins *in vivo*, which function as carriers and increase the apparent solubility of hydrophobic drugs [30]. It is thus becoming commonplace to include albumin within *in vitro* assay systems to mimic this effect [31]. R123 has previously been found to be highly bound to plasma proteins, with an unbound fraction (fu) of 0.3 in rats [20]. We, therefore, examined the propensity for R123 to bind albumin *in vitro*, using the shift in λmax associated with protein-drug interactions [30]. The change in fluorescence intensity of 0.1 µM R123 was determined for increasing albumin concentration (figure 3d), fitted using a one site total saturation model, generating a Kd = 2.2±0.3 µM. The use of albumin binding data is becoming standard for the in vitro-in vivo extrapolation of drug disposition and pharmacokinetics. It is well established that clearance rates estimated from in vitro-derived transport rates alone are often an under-prediction, often caused by binding to plasma proteins such as albumin [32]. It is thus important to derive albumin binding data for both in vitro-in vivo extrapolation (IVIVE) and to allow correction of transporter data undertaken in serum-containing medium.

As previously reported, R123 is highly bound to serum in vivo, approximately 70% in rats [20], and we demonstrate herein that this interaction has a Kd of approximately 2 µM. The concentration of albumin in serum is approximately 60–70 µM, meaning that a standard cell culture medium containing 10% calf serum will have a concentration of approximately 6–7 µM. Such a concentration will allow significant R123 binding to albumin that will, therefore, impact upon the free extracellular concentration of R123. To avoid such issues, it is recommended that tests are carried out in non-serum containing media, such as HBSS.

### Carrier-protein dependent transport of R123 across biological membranes

**Figure 4:**
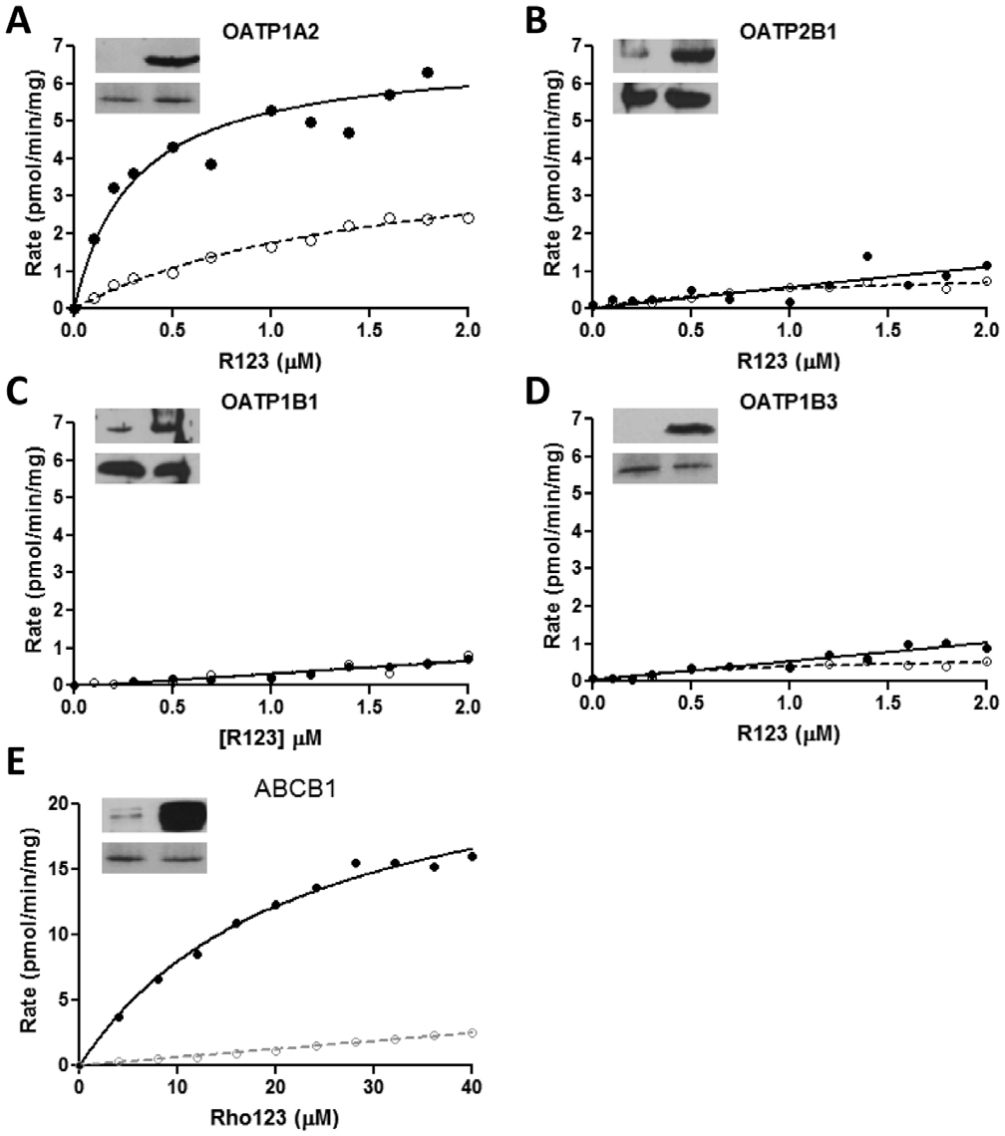
Carrier-protein dependent transport of R123 through biological membranes. (A–D) HEK-293 MSRII cells were singly transduced with BacMam viruses carrying human CDS' for OATP1A2, OATP1B3, OATP2B1 and OATP1B1 and incubated for 48 hours to allow protein expression. Cells were exposed to 0–2 µM R123, in the presence (open circles) or absence (closed circles) of 10 nM specific inhibitor for 120, 300, 420 and 600 seconds at 37°C, and intracellular fluorescence determined: Inhibitors were ketoconazole (OATP1A2), rifamycin (OATP1B1/3) and montelukast (OATP2B1). Data points correspond to the rate of uptake obtained from linear regressions over three time points with duplicate values at each concentration. (E) Naive MDCKII cells (open circles), or MDCKII cells with the CDS for human ABCB1 stably integrated (closed circles) were loaded with varying concentrations of R123 and efflux measured over 7.5 minutes. For all panels, inset images represent Western blot analysis for naïve and transfected cells (top row), plus actin loading control (bottom row), demonstrating high-level expression of the requisite human transporter in each case.

In addition to passive diffusion of R123 micelles across biological membranes, protein-mediated membrane transport may play a role in R123 disposition. R123 uptake in sandwich rat hepatocytes has been shown to be a saturable process that can be inhibited by Oatp1a4 substrates (digoxin, quinine, *d*-verapamil, 17β-estradiol-D-17β-glucuronide) [33], while the utility of R123 as an MDR1 substrate is the subject of the current study. We first examined the potential for active uptake or R123 by OATP transporters. HEK293 MSRII cells were singly transduced with human SLCO genes using BacMam technology, and R123 uptake examined in the presence of absence of appropriate chemical inhibitors. Figure 4a–d demonstrates that while R123 is a clear substrate for OATP1A2-mediated transport (Km = 0.3±0.1 µM, Vmax = 6.8±0.5 µM), it is not a substrate for the other major OATP isoforms in human liver, namely OATP1B1, 1B3 and 2B1. Such data is consistent with the uptake of R123 by Oatp1a4 in the rat, which is the orthologue of human OATP1A2 [33].

To characterise the transporter-mediated efflux of R123, we next measured the ABCB1-mediated efflux of R123 in MDCKII cells into which the coding sequence (CDS) encoding the human ABCB1 had been stably integrated. Compared to naive MDCKII cells, those expressing human ABCB1 were able to efflux R123 at a significantly faster rate (Figure 4e): This efflux was inhibited by the ABCB1 competitive inhibitor quinidine (data not shown). Subtraction of the efflux rate in naive cells from those in ABCB1-expressing cells provided a Michaelis-Menten constant (Km) of 17.5±2.8 µM and Vmax = 20.4±1.4 µM for the ABCB1-mediated active transport of R123.

R110 is known to not act as a MDR1 substrate [34], and hence the formation of this metabolic product within cell systems should not impact upon R123-mediated efflux through competitive mechanisms; there will obviously be an impact in terms of reduced R123 concentration in such metabolically active systems, but as addressed in the next section, this can be accounted for. With regard to the uptake transporters, little information is available on the ability of R110 to act as a substrate of the proteins; however, given that R110 will be formed within the intracellular compartment, while the uptake transporters derive substrate from the extracellular compartment, and that R110 passive diffusion across membranes is low, it is highly unlikely that any significant effect will be observed. As OATP1A2 can mediate the uptake of R123, a pertinent question is whether this will confound measurements of MDR1-mediated efflux of R123 *in vitro*. During the loading phase of any experiment, OATP-mediated uptake will have little impact, as extracellular concentration is maintained above the CMC, meaning passive uptake is the predominant route. However, during measurement of MDR1-mediated efflux it could be questioned whether OATP-mediated uptake into the cells could confound measurement, effectively causing an underestimation of efflux rate. Given the large external medium volume (2 mL) compared to the internal cellular volume (∼2 pL per cell or ∼0.4 uL/well) used in the efflux assay, effluxed R123 is subject to a five thousand-fold dilution. Due to this, the concentration of R123 in the assay medium is unlikely to achieve concentrations sufficiently high for OATP1A2-mediated uptake to become significant, especially when one considers that the Km for this uptake is 0.3 µM.

It should be noted that R123 may be a substrate for other uptake transporters, and has recently been reported as an OCT1/2 substrate [35]. However, given the large number of SLC and ABCC drug transporters per species (>400), it is unfeasible to test all of these for their ability to transport R123/R110. They should however, be considered potential confounding factors in any analysis. It is recommended that experimental design accounts for such confounding, with the use of over-expression systems and/or loading concentrations over the CMC, as appropriate, being possible wasy to mitigate/minimise such effects.

### Metabolism of R123

R123 is metabolised by intracellular esterases to R110 (figure 1; [5]), which is also a fluorophore, albeit with different spectroscopic properties (figure 2). As R110 is subsequently glucuronidated to become a substrate for MRP2 [21], R123 has been suggested as a dual marker for MDR1- and MRP2-mediated transport [36]. It is important to derive kinetic parameters for this metabolic conversion, such that accurate estimations of intracellular R123 concentration can be made: This, in turn, will allow the robust determination of R123 transport kinetics. We used the metabolic (S9) fraction from both MDCKII and human liver cells to determine these kinetics, while keeping the R123 concentration below the CMC (2 µM) to ensure that rate plateauing was as a result of maximum enzyme velocity and not R123 aggregation into micelles. Initial rates of fluorescence loss were correlated against R123 concentration and fitted with a sigmoidal Hill slope on the basis of Akaikes information criterion, which confirmed the selection of the allosteric sigmoidal curve fit over a Michaelis-Menten model with 99% probability [37]. R123 biotransformation in the MDCKII cell line occurred at a higher rate (Vmax = 154.6±15.51 pmol/min/mg) and with higher enzyme affinity for the substrate (K′ = 0.048±0.053 µM) than was seen when human liver-derived S9 was used (Vmax = 136.5±8.6 pmol/min/mg, K′ = 0.32±0.08 µM) (figure 3e). For both reactions, the Hill slope (h) was approximately 2, suggestive of positive cooperativity upon substrate binding to one or more binding sites on the enzyme(s). Whilst it is difficult to accurately assess the metabolism rate of R123 in cells, due to the large number of factors that influence this rate, it is possible to reach a reasonable estimation based upon several assumptions, such as the volume of cells, their protein content, the equilibration between intracellular R123 pools etc. Based upon these assumptions, we estimate that at Vmax rates, metabolism of R123 in vitro would consume approximately 1% of the R123 pool every minute. This is likely an overestimation, as Vmax would not always be achieved/maintained, but even under such conditions, less that 10% of the R123 would be lost through metabolism during the 7.5 minute experimental period.

### Comparison of R123 to other tracer dyes

R123 is not the only chemical that has been proposed for use as a tracer dye for MDR1. The most commonly used alternatives are currently Hoechst 33342 [38], 3,39-diethyloxacarbocyanine iodide and, calcein-AM [39], with commercially systems such as eFluxx-ID also being recently released (Enzo Lifesciences; [40]). However, it should be noted that Prochazkova and colleagues recently demonstrated that dihydrorhodamine 123, dihexiloxocarbocyanine iodide, hydroethidine, tetrachloro-tetraethylbenzimidazolocarbo-cyanine iodide and tetramethylrhodamine ethyl ester perchlorate were all substrates for MDR1, and hence could represent potential tracer dyes [41]. It should be noted that all of these dyes have the potential limitation that they possess liabilities for transport by other members of the ABC and SLC superfamilies of membrane transport proteins. Such transport may confound measurement of MDR1-mediated activity unless suitable controls are employed (e.g. the use of overexpression systems or specific pharmacological inhibitors to allow the derivation of the MDR1-component for any efflux. As noted herein, access of lipophilic tracer dyes, including R123, into the cell interior may be a limiting factor to their utility. A solution to this is the modification of the tracer dye to a membrane-permeable precursor, as seen with the hydrophobic ester derivative calcein acetoxymethyl (Calcein-AM): once in the cell these esters are rapidly cleaved to reveal the fluorescent probe substrate [40]. However, as described herein, simple derivation of the CMC for R123 allows efficient loading of cell without the need for derivatisation of the base fluorophore; indeed this is an approach that we would propose as a general first step in optimising tracer dye use. One advantage of R123 over tracer dyes such as Hoechst 33342 is its fluorescent stability, with R123 showing very little photodegradation over time (data not shown compared to Hoeschst 33258, which is liable to both photodegradation and quenching [42].

### Conclusion

Tracer dyes are commonly used as biologically-silent probes to examine processes within the cellular context. Their low cost, good availability and ease of measurement make them ideal for use in both high throughput and high content biological studies. In order to maximise data from these studies, it is important to fully characterise the spectral properties of these tracer dyes, plus their interaction with the cellular environment. Herein, we confirm the λmax for R123 excitation and emission, and demonstrate that these are altered significantly by local chemical environment, with 1% (v/v) methanol in HBSS providing the best compromise of solubility, quantum yield and cellular toxicity. In addition, we demonstrate that R123 has a CMC of 1.9 µM, meaning that cellular loading is rapid at concentrations above 2 µM. Finally, we demonstrate that R123 is rapidly distributed within cells, partitioning into subcellular membranes and organelles, as well as being metabolised into R110, a related flurone. These data allow an accurate estimation of the sub-cellular pharmacokinetics of R123, an important step in its optimisation as a tracer dye.

## Materials and Methods

### Cell Lines and Routine Maintenance

The human hepatocellular carcinoma (Huh7) cell line is a differentiated, immortalised cell line commonly used as an *in vitro* hepatocyte model [43], [44], and was a kind gift of GlaxoSmithKline. Madin-Darby canine kidney cells, strain II (MDCKII) share some characteristics with both proximal and distal tubule cells [45], are commonly used as a polarising cell line for transporter studies, and were purchased from ECACC (ECACC code 00062107, Porton Down, UK). Both cell lines were cultured in Dulbecco's modified eagle medium (DMEM) with L-glutamine and phenol red, containing 10% foetal bovine serum, 1% non-essential amino acids (NEAAs), 100 U/ml penicillin and 100 µg/ml streptomycin. All cell culture reagents were purchased from Invitrogen (Paisley, UK).

Human Embryonic Kidney (HEK; ECACC code 85120602) are derived from healthy human embryonic kidney and later modified to express human Class A macrophage scavenger receptor (HEK293-MSRII) to improve cell adherence [46], and were a kind gift of GlaxoSmithKline. Cells were cultured in DMEM:Hams F12 nutrient mixture (1∶1) with L-glutamine and phenol red, containing 10% foetal bovine serum and 0.4 mg/ml Geneticin.

For all cell lines, cells were routinely cultured in 75 cm^2^ growth area tissue culture flasks, and passaged when they reached approximately 80% confluence. To ensure phenotypic consistency throughout experiments, each cell line was only used for a fixed number of passages after recovery from storage: Huh7 cells were used up to 12 passages after receipt, while MDCKII and HEK293-MSRII cells were used up to passage 54 from a new stock.

### Cell lysis Solutions

For the determination of cell lysis solution impact on R123 fluoresence we prepared three commonly used cell lysis solutions: RIPA buffer (50 mM Tris.HCl pH7.4, 150 mM NaCl, 2 mM EDTA, 1% nonidet p40, 0.1% SDS), PEB (2% nonidet p40, 0.2% SDS, 1 µM dithiothrietol) and 2% Trition ×100.

### Facilitated transport capacity using BacMam Transduced HEK293-MSRII Cells

For active uptake experiments, cells were transduced with BacMam virus (Invitrogen) containing the coding sequence for each of the four major SLC transporters in human liver; namely, OATP1A2, OATP1B1, OATP1B3, and OATP2B1. The method of transduction, maximum permitted percentage of BacMam, plaque forming units (pfu) per millilitre and the multiplicity of infection (MOI) were optimised for each expression plasmid in pilot experiments, as summarised in Table 1. In all cases, BacMam reagent was kept protected from light at 4°C, and sodium butyrate (2 mM) added to medium prior to BacMam to increase transduction efficiency, and then transduction undertaken as per the manufacturer's instructions. For OATP1B1 in well-transduction, cells were plated at 2×10^5^ cells/cm^2^ and allowed to adhere for 4 hours prior to removal of medium and addition of BacMam, whilst for in-tube transductions, cells were transduced and then plated at the concentrations indicated in table 1. All transduced cells were placed in a humidified incubator at 37°C with 5% CO_2_ for 48 hours prior to uptake analysis to allow expression and correct localisation of the encoded SLC transporter.

**Table 1:** Optimal conditions for BacMam transduction of transporter expression plasmids in HEK293-MSRII cells.

Transporter	Mode of Transdution	Pfu/ml	Final Seeding density (cells/cm^2^)	MOI	Max BacMam (%)
OATP1A2	In-tube	1.07×10^8^	2.6×10^5^	60	14
OATP1B1	In-well	1.78×10^8^	2.1×10^5^	180	20
OATP1B3	In-tube	1.54×10^8^	2.1×10^5^	155	20
OATP2B1	In-tube	6.80×10^8^	2.6×10^5^	40	1

Pfu = plaque forming units; MOI = multiplicity of Infection.

Following transduction, uptake assays were undertaken as follows: Medium was removed and cells washed with prewarmed 37°C Dulbecco's phosphate buffered saline (DPBS; Invitrogen). 0–2 µM R123 (1% methanol, DPBS) (Sigma-Aldrich, Poole, UK) was added per well, and incubated in a humidified incubator at 37°C. At the end of the timed incubation, solutions were removed from the wells, followed by the immediate addition of ice-cold DPBS to each well. Cells were washed a further two times with ice-cold DPBS before fluorescence was measured; intracellular R123 concentration was then determined against a R123 standard curve prepared in the same medium. For each transduction experiment, transporter functionality was determined by uptake of radiolabelled model substrates (estradiol 17β-D-glucoronide for OATP1B1/3 and estrone sulphate for OATP1A2 and 2B1), and the inhibition of transport activity by specific SLC-inhibitors (10 nM ketoconazole for OATP1A2; 10 nM rifamycin for OATP1B1/3; 10 nM montelukast for OATP2B1).

### Intracellular Accumulation of R123 in MDCKII/MDCKII-ABCB1 Cells

Cells were initially plated at 2×10^5^ cells per cm^2^ in 24-well plates and allowed to reach 80% confluence. Cells were then exposed to 0–10 µM R123 (1% methanol, HBSS) for 10 minutes, followed by washing three times with ice-cold DPBS to remove excess R123, and lysis with 2% (v/v) triton-×100 in HBSS with 1×protease inhibitors (Roche Diagnostics, Burgess Hill, UK). The lysate was then diluted 1∶2 with HBSS and the fluorescence determined against a standard curve prepared by spiking R123 into cell lysate with 2% (v/v) triton ×100.

### Efflux Assays with R123 in MDCKII/MDCKII-ABCB1 Cells

MDCKII and MDCKII-ABCB1 were initially plated at 2×10^5^ cells per cm^2^ in 6-well plates and allowed to reach 80% confluence. Cells were then exposed to 0–20 µM of R123 (1% methanol, HBSS) for 10 minutes at 37°C, to allow loading of R123 into cells. Following loading, cells were washed three times with cold PBS to remove excess R123, and efflux initiated by the addition of 3 ml HBSS pre-warmed to 37°C. R123 fluorescence was determined after 7.5 minutes, a time previously determined to be within the linear phase of the assay. Cells were subsequently lysed with 2% (v/v) triton-×100 in HBSS with 1×protease inhibitors, total protein measured by the Lowry method [47], and R123 concentration normalised to protein content. Finally, R123 concentration was determined against a standard curve measured under the same conditions.

### Metabolism of R123 to R110

R123 is metabolised to R110 by the removal of a methyl ester group [48]. R110 is also a fluorophore, but with a lower quantum yield for any given excitation wavelength. Thus, the rate of conversion of R123 to R110 can be determined by measuring the fluorescence decrease over time. Metabolism was measured in HBSS supplemented with 5 mM MgCl_2_, 5 mM glucose-6-phosphate, 0.5 mM β-NADP^+^ and 0–1.4 µM R123 (1% methanol, HBSS), as indicated. The reaction was initiated by addition of MDCKII- or human liver-derived S9 fraction at a final concentration of 0.1 mg/ml and 1 mg/ml, respectively, and fluorescence decrease over 2 minutes measured.

S9 fractions were prepared from either human liver or MDCKII cells. Scissor-minced human liver in 1.15% KCl, or MDCKII cells in DMEM were initially homogenised using either a Dounce homogeniser or 21 guage needle, respectively. Cell suspensions were then centrifuged for 5 minutes at 800×*g*. Medium was removed and the pellet resuspended in cold HBSS. Cells were then homogenised and lysed by passing ten times through a 21 gauge needle. This lysate was then centrifuged for 20 minutes at 9000×g at 4°C and the supernatant (containing microsomes and soluble fraction) assayed for protein concentration using the Lowry method [47].
